# A Simple Threshold Rule Is Sufficient to Explain Sophisticated Collective Decision-Making

**DOI:** 10.1371/journal.pone.0019981

**Published:** 2011-05-24

**Authors:** Elva J. H. Robinson, Nigel R. Franks, Samuel Ellis, Saki Okuda, James A. R. Marshall

**Affiliations:** 1 School of Biological Sciences, University of Bristol, Bristol, United Kingdom; 2 York Centre for Complex Systems Analysis, Department of Biology, University of York, York, United Kingdom; 3 Department of Computer Science and Kroto Research Institute, University of Sheffield, Sheffield, United Kingdom; University of Utah, United States of America

## Abstract

Decision-making animals can use slow-but-accurate strategies, such as making multiple comparisons, or opt for simpler, faster strategies to find a ‘good enough’ option. Social animals make collective decisions about many group behaviours including foraging and migration. The key to the collective choice lies with individual behaviour. We present a case study of a collective decision-making process (house-hunting ants, *Temnothorax albipennis*), in which a previously proposed decision strategy involved both quality-dependent hesitancy and direct comparisons of nests by scouts. An alternative possible decision strategy is that scouting ants use a very simple quality-dependent threshold rule to decide whether to recruit nest-mates to a new site or search for alternatives. We use analytical and simulation modelling to demonstrate that this simple rule is sufficient to explain empirical patterns from three studies of collective decision-making in ants, and can account parsimoniously for apparent comparison by individuals and apparent hesitancy (recruitment latency) effects, when available nests differ strongly in quality. This highlights the need to carefully design experiments to detect individual comparison. We present empirical data strongly suggesting that best-of-n comparison is not used by individual ants, although individual sequential comparisons are not ruled out. However, by using a simple threshold rule, decision-making groups are able to effectively compare options, without relying on any form of direct comparison of alternatives by individuals. This parsimonious mechanism could promote collective rationality in group decision-making.

## Introduction

Animals need to make choices between multiple available options at many stages in their life histories, such as during mate selection, foraging, or when deciding where to shelter or to build a nest. The fitness benefits of choosing the best option mean that decision-making strategies will be subject to natural selection. Multiple comparison or ‘best of n’ strategies perform most accurately, and are likely to be optimal when searching and sampling costs are low, e.g. on a lek [1], [2] and when long-term fitness implications of the decision are high, e.g. in home range selection [3]. In other situations, e.g. during foraging, there can be substantial time costs to making accurate decisions [4], [5], and these costs can be so great as to make quicker less accurate decisions more efficient [6]. Animals may be best served by using a simpler ‘rule of thumb’, which reduces sampling time, but still ensures the option they choose is good enough. One simple but effective strategy is sequential search in which the animal keeps searching until it finds an option that exceeds a threshold of acceptability [2], [7]. This kind of fixed-threshold strategy is used in foraging [8], mate choice [9] and refuge selection [10]. An intermediate strategy is to allow thresholds to be influenced by experience of previous options; in effect this would cause a ‘sequential comparison’.

Social animals make collective decisions between available options, particularly about where the group spends its time. When making a shared collective decision, a group faces the challenges of integrating information from multiple individuals and managing the different decision-preferences of group members [11]. Such decisions are influenced by individual information, conflicts of interest and time-constraints [12], [13]. To understand how a collective decision is reached, the rules followed by contributing members of the group must be identified [14]. Emigrating social insects are a good model system for the study of collective decision-making, because all members share the aim of coming to a consensus about a new, good nest site as soon as possible, but the information about available site quality is distributed very unevenly within the colony [15], [16]. To investigate the mechanism of decision making, we used models of, and experiments on, emigrating cavity-nesting rock ants, *Temnothorax albipennis*.

**Figure 1:**
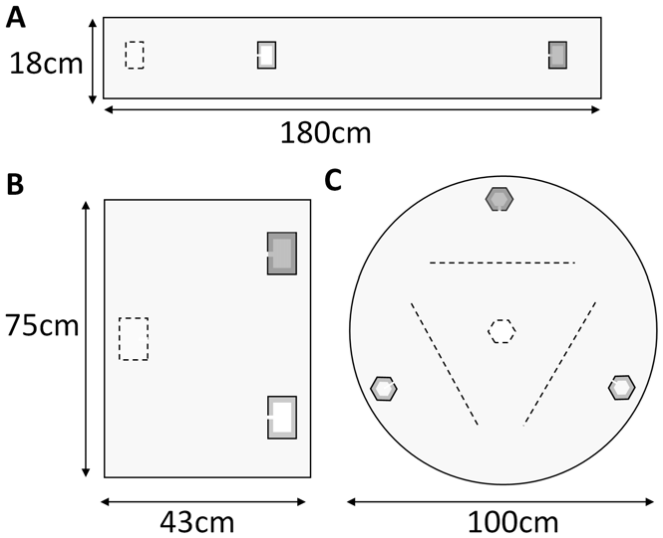
Arenas used in emigration experiments, showing nest locations. Dashed outline = old destroyed nest; shading = good nests. (A) Good nest 120 cm from old nest; poor nest 30 cm [26]. (B) Equidistant good and poor nests 45 cm from old nest [20]. (C) Three equidistant new nests 36 cm from old nest. Dashed lines indicate points at which tandem-runs were recorded.

A colony emigration by rock ants begins with scouts searching for and assessing new sites. Successful scouts recruit nest-mates using tandem running, in which an informed ant leads another to the new site [17]. When the number of ants at a site reaches a ‘quorum threshold’, the ants switch to rapid transport behaviour to carry the brood, queen and remaining nest-mates to the new nest [18]. Ant colonies discriminate between nest sites on the basis of a range of attributes including cavity dimensions, light level and entrance width [16], [19]. Three possible mechanisms by which the colony collectively chooses the better of two nests have been proposed: i) comparison ii) recruitment latency iii) threshold rule. First, ants could use a comparison strategy, in which ants that have visited both nests would compare their qualities and recruit nest-mates only to the better one [20]. Second, ants visiting just one nest could determine how long they hesitate before recruiting nest-mates (recruitment latency) based on the nest quality [20]. A combination of these two mechanisms has been used as the basis of several decision models [18], [21]–[24]. Although lone *T. rugatulus* ant workers are capable of comparing the attributes of nest-sites which are very close together [25], the evidence for individual ants making direct comparisons between nests during colony emigration is weak [26], and furthermore, ant colonies are able to choose a distant good nest over a nearby poor nest, when recruitment latency differences would be expected to be cancelled out by travel time (Fig. 1a) [26], [27]. The third possible mechanism of choice is a sequential search strategy, based on a very simple rule of thumb, a ‘threshold rule’, by which an ant assesses a nest against her own fixed quality threshold, and either accepts the nest and begins recruitment, or rejects it and continues searching [26]. Quality-dependent recruitment latency has been demonstrated empirically only when colonies were presented with a single new nest and were not required to make a choice [20], [22], [24], [28]. Robinson *et al.* [26] hypothesized that these apparent recruitment latency effects could emerge as a by-product of a threshold rule, because ants that find a low-quality nest will tend to reject it and continue searching, whereas ants that find a high quality nest will tend to accept it and begin recruitment. In addition, Robinson *et al.* [26] hypothesized that the apparent comparison phenomenon in which ants that have visited equidistant poor and good nests usually recruit only to the good nest (Fig. 1b; [20]) can be explained more parsimoniously with a threshold rule in which the poor nest is rejected and forgotten, then the good nest is discovered and accepted, rather than requiring individual ants to perform the more cognitively complex task of remembering and comparing the qualities of different nests. We aim to test the hypothesis that this simple parsimonious mechanism (the threshold rule) is sufficient to reproduce observed empirical patterns of collective decision-making.

Here we present a Markov-chain model of decision-making, based on a simple threshold rule. Our model ants search for nests, and compare those they find to an internal acceptance threshold, subject to assessment error. This model includes no memory of previous nests, and no ability to make direct comparisons between the nests an ant has encountered. It also includes no explicit quality-dependent recruitment latency. We compare the results of this model to empirical results from two published studies and one new experiment, to test whether our model can produce the qualitative patterns seen in the empirical data. By parameterizing our model as far as possible to previous experiments [20], [26] we test whether this simple threshold-rule is sufficient to explain apparent recruitment latency and comparison effects. We also use the model to predict emigration behaviour in a new experiment that presents an ant colony with three equidistant nests, one good and two poor (Fig. 1c). We performed this experiment using individually radio-tagged ants to monitor individual-level behaviour and compare our empirical results to the model predictions. We thus test whether a very simple individual-level ‘rule of thumb’ is sufficient to explain sophisticated collective decision-making.

## Methods

### Model

**Figure 2:**
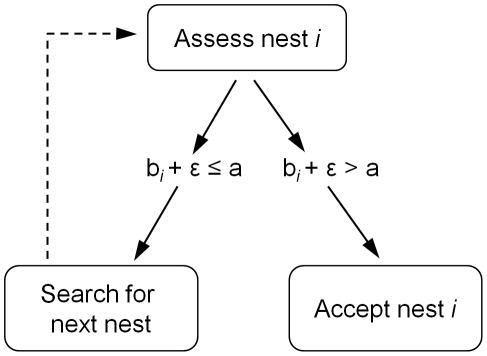
Schematic of model. A simulated ant continues searching until it encounters a nest of a quality (*b*) exceeding the ant's individual threshold (*a*), taking into account assessment error (*ε*). Ants may revisit the same nest (with probability *r*), and do not have any memory of previously visited nests.

To investigate the proposed threshold rule, we present a very simple model. As we are only concerned with independent discovery by scouts before recruitment commences, we examine the colony's decision process analytically and using Monte-Carlo simulation, modelling ants as independent instantiations of a Markov process. Ants independently accept sites according to a probability specified by the site's quality and their individual threshold, otherwise they search randomly for a site to assess (Fig. 2). We have modelled a discontinuous acceptance function here, but this could be relaxed to be a smooth (e.g. sigmoidal) function of difference between internal threshold and sampled quality, without qualitatively affecting the results. The Markov process has five states: ‘assessing home site’, ‘assessing poor site’, ‘assessing good site’, ‘committed to poor site’, and ‘committed to good site’. All individuals start in the ‘assessing home site’ state but the home site is considered uninhabitable in the model, and its quality is set to negative infinity, therefore an ant can never become committed to it. Ants can switch between the assessment states, but we assume that once committed to a site an ant remains so and recruits nest-mates to its preferred option; the recruitment process is not modelled, and the ‘committed’ states are therefore absorbing states.

For the Monte-Carlo simulations, we model nest quality acceptance thresholds in the colony as a normally distributed random variable *A*, with some mean and variance. An individual simulated ant has a quality acceptance threshold *a* drawn at random from this distribution. It discovers its next nest by sampling from a probability distribution specified by its current nest, arena size and shape, and the proximities of other available nest sites (Table 1); part of this distribution is determined by the probability *r* that a randomly searching scout rediscovers the site it has just been in. On discovering a site, the scout evaluates the quality of the current nest site with some error *ε* added, sampled from a standard normal distribution (mean 0; standard deviation 1); this corresponds to error in the scout's quality assessment. If this sampled quality exceeds the scout's acceptance threshold then it becomes committed to the site, otherwise it continues searching (Fig. 2). Acceptance threshold distributions and nest qualities are given arbitrary values (Table 1), because individual probabilities of becoming committed to a nest are hard to estimate empirically, due to the difficulties in accurately identifying commitment and in eliminating the effects of interactions between ants. In the model, travel times between sites are sampled from normal distributions. By parameterizing this model to empirical ant movement speed and specific experimental arenas (Table 1), we can approximate the expected time for each simulated ant to find each nest, and the time from first finding a nest to becoming committed to it (the ant may visit other nests in between, or visit the same nest many times before committing to it). This allows us to calculate recruitment latencies for comparison with empirical data. In the empirical studies, recruitment latency concerns the time from discovering a nest to first recruiting to that nest. We do not include recruitment in the model, so modelled recruitment latency concerns the time from discovering a nest to ‘accepting’ that nest. For real ant colonies, searching behaviour is much reduced when quorum is achieved; we truncate the simulated data at the time at which quorum was achieved in the real experiment, to increase comparability (see Text S1).

**Table 1:** Parameterisation used in simulations of Monte-Carlo model.

Parameter	Comparison with [26]	Comparison with [20]	Comparison with new multiple-nest experiment	Derivation
Number of nests	3	3	4	From experiments
Arena size and shape	See Fig. 1a	See Fig. 1b	See Fig. 1c	From experiments
Position of nests	Good nest (A) further than poor nest (B) (Fig. 1a)	New nests equidistant from old (Fig. 1b)	New nests equidistant from old (Fig. 1c)	From experiments
Mean travel time between nests (sec) from column nest to row nest (SD = 1/5 mean)	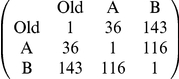	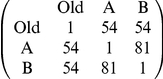	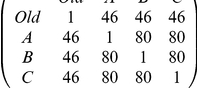	From walking speed 8.4 mm/s [74]
Probabilities of finding nests (from column to row)^1^	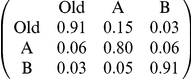	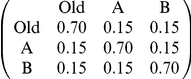	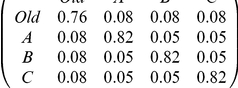	From arena size, arena shape & nest positions; see Text S1.
Number of ants	27 (Fig. 3); 49 (Fig. 4)	29 (Table 2); 12–63 (Fig. 5)^2^	13 (test 1); 20 (test 2)	From experiments
Acceptance threshold distribution (A)^3^	Normal distribution:mean = 5, SD = 1	Normal distribution:mean = 5, SD = 1	Normal distribution:mean = 5, SD = 1	Arbitrary
Nest qualities (b)^3^	Old = −inf; Poor = 4; Good = 6	Old = −inf; Poor = 4; Good = 6	Old = −inf; Poor = 4; Poor = 4; Good = 6	Arbitrary
Assessment error (from which ε is drawn)^3^	Normal distribution:mean = 0, SD = 1	Normal distribution:mean = 0, SD = 1	Normal distribution:mean = 0, SD = 1	Arbitrary

1 This includes *r*, the probability of re-discovering the same nest.

2 Numbers of ants are colony specific for recruitment latency simulations; see Fig. 5.

3 These acceptance threshold distributions and error rates correspond to quality-dependent nest acceptance probabilities of 0.76 for the good nest and 0.24 for the poor nest. See Text S1 for details.

#### Model Analysis

In addition to the simulation model described in this paper, we can derive analytic results for the proposed rule for switching between nests. To facilitate this analysis we simplify the Markov process slightly from that used in the Monte-Carlo simulations, by discarding the state of assessing the home site. The four states in the simplified Markov process are thus ‘assessing poor site’, ‘assessing good site’, ‘committed to poor site’, and ‘committed to good site’, and the state transition matrix is (with states ordered as described above from top to bottom and from left to right)
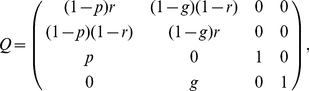
(1)where *p* is the average per-visit probability that an ant accepts the poor site, *g* is the average per-visit probability that an ant accepts the good site, and *r* is the probability that a randomly searching scout rediscovers the site it has just been in. These are colony-wide average probabilities that can easily be derived from the threshold *A* and error *N*(0,1) distributions used in the Monte-Carlo simulation, if comparability is desired. The matrix *Q* in eq. 1 can be understood as having a column corresponding to each state in the model, where that column specifies a probability distribution over the next state. The probability distribution over the initial states (‘assessing poor site’ and ‘assessing good site’) can be derived based on the relative probabilities of an arbitrary ant scout discovering either when searching from the home site.

Using standard techniques [e.g. 29] we derive analytic results on the long-run behaviour of the threshold-switching rule, using the state transition matrix *Q* (eq. 1) and its fundamental matrix. Of particular relevance to our proposals is the analysis of the expected time for the Markov-process to reach *any* absorbing state, when started in some particular non-absorbing state. If we assume both candidate nest sites are equally likely to be discovered first from the home site, then the expected number of steps in the Markov-chain before commitment to *either* nest site is reached is given by
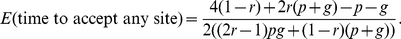
(2)Equation (2) will be used below to examine the sensitivity of acceptance time to changes in site qualities. Note that, technically, we should be interested in the latency between discovering a particular site, and recruiting to that site, however in general per-state latencies are difficult to calculate in Markov-chains, and the overall expected latency given in equation 2 is an acceptable simplification (see Text S1).

### Empirical methods

#### Ethics statement

Our experiments on *T. albipennis* and collection of colonies from the field comply with the laws of the country in which they were carried out and the relevant national and international guidelines [30], [31].

#### ‘Three-New-Nests’ Experiment

Six *T. albipennis* colonies were collected from the Dorset coast, England, June–July 2009. Colonies were queenright and contained 80–200 workers and brood of all stages. Colonies were housed in artificial nests [27] and provided with water *ad libitum* and honey solution and *Drosophila melanogaster* weekly. We used these colonies to investigate decision-making when a colony was offered a choice of three new nests. Six trials were performed, each with a new colony. One day prior to an emigration trial, we attached an RFID microtransponder (500×500×120 µm) with a unique ID to the thorax of every worker ant in the colony [32], [33] and housed the colony in a nest formed of a hexagonal glass slide (edge length = 30 mm), a 1.5 mm cardboard perimeter (cavity area 1496 mm^2^) and an acetate lid with a central entrance hole (1.5 mm diameter) to avoid introducing directional bias. We performed trials in a circular arena with Fluon-coated sides, the floor of which was cleaned with water and alcohol between trials. Three new hexagonal nests with the same dimensions as the original nest were placed in the arena (Fig. 1c). Two nests had clear acetate lids and one had a red filter covering the cavity area, making the nest appear relatively dark to the ants [34]. Ant colonies choose dark over light nests [19]. The position of the good nest was interchanged between trials. The new nests had 1.5 mm wide entrances in the side facing the centre of the arena, over which RFID readers (PharmaSeq, Inc., NJ) were placed vertically. A trial began with the original nest placed in the centre of the arena and then destroyed by removing the lid. The RFID readers detected ants entering and leaving the new nests [86% tag read rate, 26] and we used handheld RFID readers to read the tags on tandem-running ants (94% tag read rate) as they crossed half-way lines (Fig. 1c). The identity of the leader and follower was thus recorded and the direction of the tandem run was noted. This procedure does not disrupt tandem runs [26]. The time at which the transport by carrying of a nest-mate or a brood item first occurred to each nest was recorded (i.e. the time at which the quorum threshold was reached). Once nest-mate transport has commenced, the RFID system becomes less reliable [26], because nest-mates are transported in such a way as to block the RFID tag. We therefore focused our analysis on searching and recruitment by tandem-running during the pre-quorum period. We observed emigrations until complete (old nest empty of brood and workers) and recorded the nest choice of the colony after 24 hours. Colonies were considered to have split if brood was present in two or more nests.

### Statistical Methods

Mean recruitment latencies were analysed using generalised linear mixed models (GLMM) ‘glmmPQL’ in the MASS library in R 2.4.1 with nest quality as a fixed factor, colony (empirical results) or replicate (simulation results) as a random factor and a Poisson error structure. Differences between recruitment latency survivorship curves, were tested using generalized log-rank tests using the function ‘survdiff’ in the survival library in R 2.4.1. Monte Carlo simulations were carried out using MatLab 7.4.0 (see Text S1), and matrix manipulation with Maple 14.

## Results

### Comparison with Poor Nest Near; Good Nest Far Data

**Figure 3:**
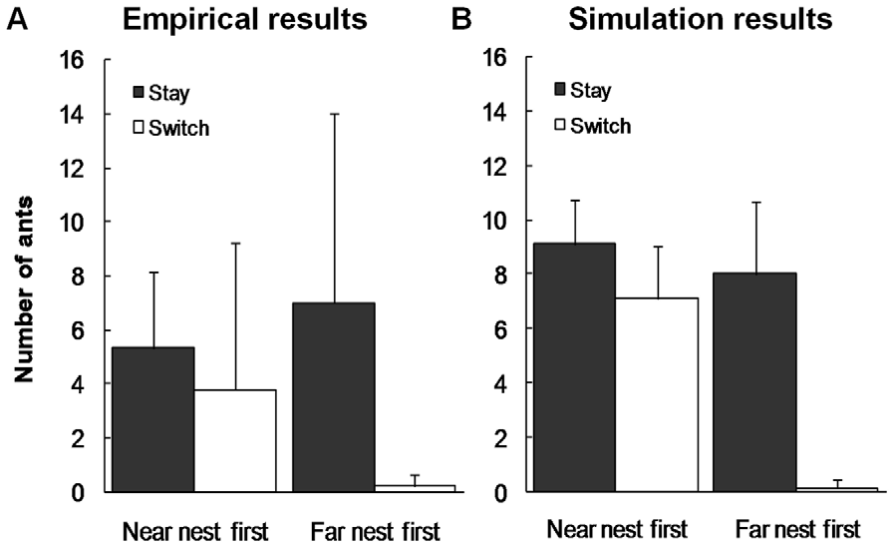
Comparison of empirical and simulated emigration behavior. Number of ants continuing to visit the same nest (stay) or going on to visit the other nest (switch), depends on the quality of the first nest visited. (A) Empirical results, 9 colonies, mean + SD. (χ^2^_1_ = 86.6, *P*<0.001), reproduced from Figure 2a, Robinson et al. [26] with permission from Royal Society Publishing. (B) Simulation results, 9 replicates, mean + SD (χ^2^_1_ = 42.0, *P*<0.001). A sample set of 9 replicates are shown here for comparability with the empirical data. Running 100 repeat sets gave the same pattern, statistically significant in 100% of cases.

We parameterized the model to the experimental set-up used by Robinson *et al.* [26] with a nearby poor nest and a more distant good nest (Table 1, Fig. 1a) and performed Monte Carlo simulations of this experiment. The switching behaviour (ants moving from visiting one nest to visiting the other) in the simulations is qualitatively very similar to the empirical data (Fig. 3). In both cases most ants that find the far (good) nest first stay at that nest, whereas ants that find the near (poor) nest first may stay or switch.

**Figure 4:**
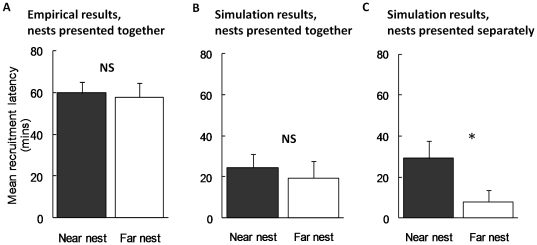
Comparison between empirical and simulated recruitment latency results where the higher quality is further away. Recruitment latencies to the near (poor) and far (good) nests, mean + SE. (A) Empirical data [26]; nests presented together, n = 9 colonies. No significant differences in recruitment latencies: GLMM: *t*_39_ = 0.08, *P* = 0.93. (B) Simulation data; nests presented together, n = 9 replicates. No significant differences in recruitment latencies: GLMM: *t*_39_ = 0.71, *P* = 0.48. Sample set of 9 replicates shown; of 100 repeat sets, 90% showed no significant difference in recruitment latencies. (C) Simulation data; nests presented separately, n = 9 replicates. Recruitment latencies to poor nest significantly greater: GLMM: *t*_88_ = 2.19, *P*<0.05. Sample set of 9 replicates shown; of 100 repeat sets, 70% showed significantly greater recruitment latencies to poor nest.

The model simulations also reproduce the empirical results that recruitment latency does not play an important role here. In the empirical study, recruitment latency was measured as time from discovering a nest to first leading a tandem run to that nest and data are only available for ants which recruited by tandem-running. There are more recruitment latencies for the simulated data, as all ants make a choice in the model, whereas in the real data not all active ants perform tandem runs. This leads to quantitative differences between simulated and empirical data, but qualitatively the patterns are the same: In the simulated data, as in the empirical data, there are no significant differences between recruitment latencies to the good and poor nests (Fig. 4a, 4b). However, in a simulation presenting the two nests separately (i.e. either only old nest and near poor nest are present, or only old nest and far good nest are present) the pattern is dramatically different, with significantly higher recruitment latencies for the poor nest (Fig. 4*c*). This clearly demonstrates that differences in recruitment latency can be generated when only one nest is present, as a side-effect of the threshold rule.

### Comparison with Two-Equidistant-Nests Data

To test whether our model can account for the patterns seen in the original experiments, used as evidence for the recruitment latency and comparison mechanisms [20], we also parameterised the Monte Carlo model to this experimental set-up (Fig. 1b, Table 1). Here two equidistant new nests, one good and one poor, are presented to the ant colony. The empirical data for direct comparison are shown in Table 2. Very similar results emerge in our simulations, without any memory or comparison coded into the model (Table 2), clearly showing that this sort of behavioural pattern can be accounted for by the threshold rule alone, without needing to include other mechanisms. However, the exact quantitative results of our simulations are influenced by the acceptance threshold and nest quality parameters. Since we do not know the shape of the real threshold distribution in an ant colony, the match between simulated and empirical data here does not prove that comparison plays no role in ant decision-making, but does show that results of this type do not necessarily have to be the produced by comparative evaluation.

**Table 2:** Empirical and simulation results for apparent comparison of nests.

	Empirical Results^b^	Simulation Results^c^
Percentage of active ants that visit both nests^a^	79%	85% (71–96)
Percentage of switching ants that recruit to good nest	92%	98% (83–100)

a Active ants are the ants that discover one or both of the new nests before quorum is reached and nest-mate carrying begins.

b Empirical results are from 3 colonies [20].

c Simulation results given as mean and 95% confidence intervals from 100 replicates.

**Figure 5:**
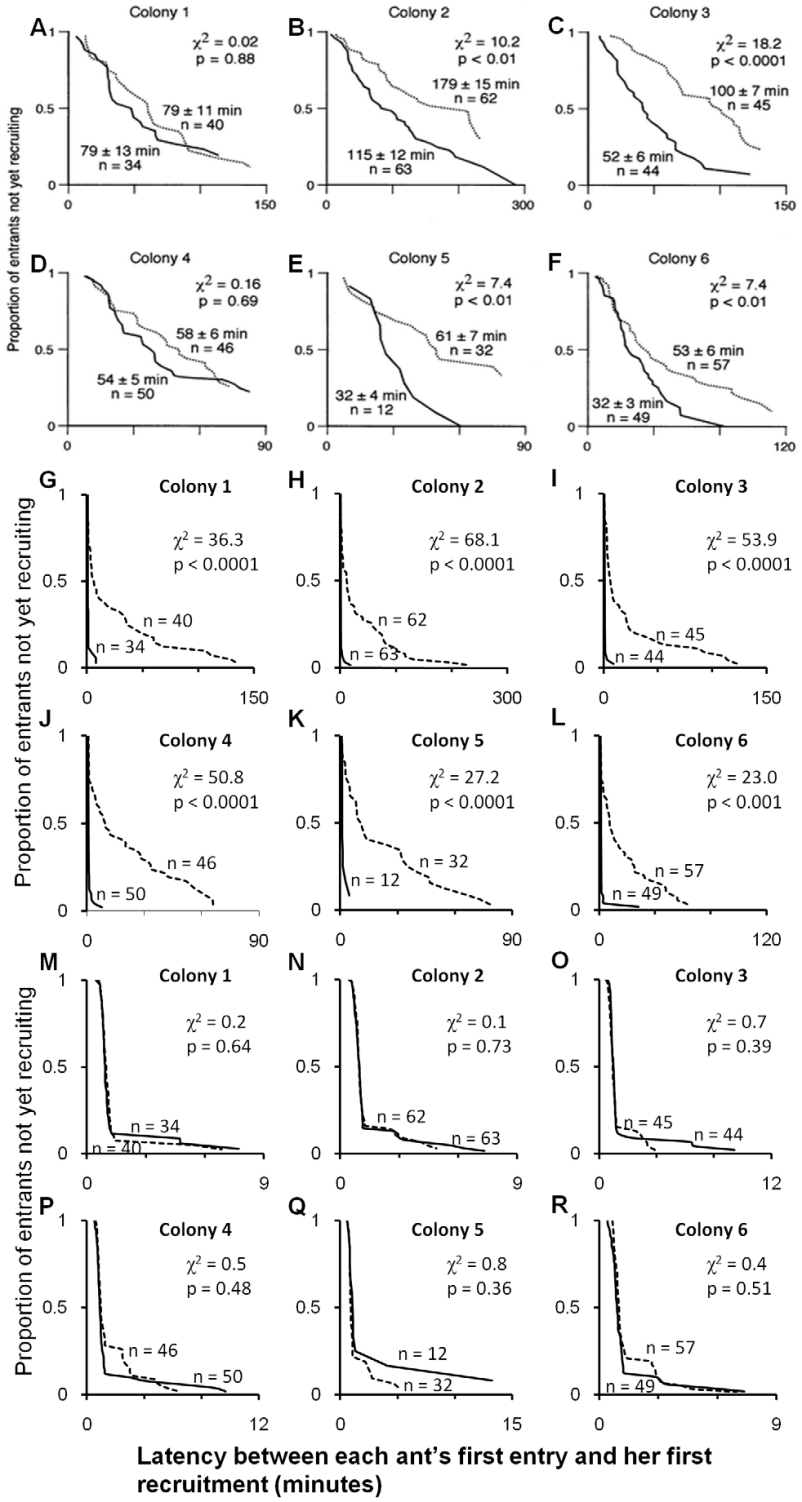
Comparison between empirical and simulated recruitment latency results where new nests are equidistant. Empirical and simulated recruitment latencies (solid line = good nest; dashed line = poor nest). The latency between entry and recruitment (mean ± SD), and the number of ants analysed, are shown next to the corresponding survivorship curve. (A–F) Empirical results with nests presented separately, reproduced from Figure 4, Mallon et al. [20] with permission of Springer Science and Business Media. Recruitment latencies to the poor nest are significantly greater in four of six colonies (generalised logrank test). (G–L) Simulated recruitment latencies, nests presented separately. Recruitment latencies to the poor nest are significantly greater. Empirical number of ants is matched for each colony. Sample graphs are shown; running 100 replicates of each gives the same pattern of results, with significant differences between recruitment latencies in 95% (Colony 5) or 100% (Colonies 1–4 and 6) of simulations. (M–R) Simulated recruitment latencies, nests presented together. There are no longer any significant differences between recruitment latencies. Empirical number of ants is matched for each colony. Sample graphs are shown; running 100 replicates of each gives the same pattern of results, with no significant difference between recruitment latencies in 96% (Colony 1), 93% (Colony 2), 90% (Colony 3) 90% (Colony 4), 96% (Colony 5) 93% (Colony 6) of simulations.

In the empirical data (Fig. 5a–f), there do appear to be quality-dependent differences in recruitment latency – however, the nests were presented separately in these experiments. Recruitment latency is measured using survival analysis, treating ants that find a site but never recruit to it as censored data. Applying the same analysis to our simulated results demonstrates that we can replicate these differences in recruitment latency when the nests are presented separately (Fig. 5g–l) but the differences disappear when the simulations are repeated identically, but with the nests presented together (Fig. 5m–r). This is same pattern as was observed in the comparison with the the ‘poor nest near; good nest far’ data, even though recruitment latencies are measured differently in the two empirical studies. These simulation results demonstrate that the observed empirical differences in recruitment latency can be explained as a side-effect of the threshold rule, when only one new nest is present.

**Figure 6:**
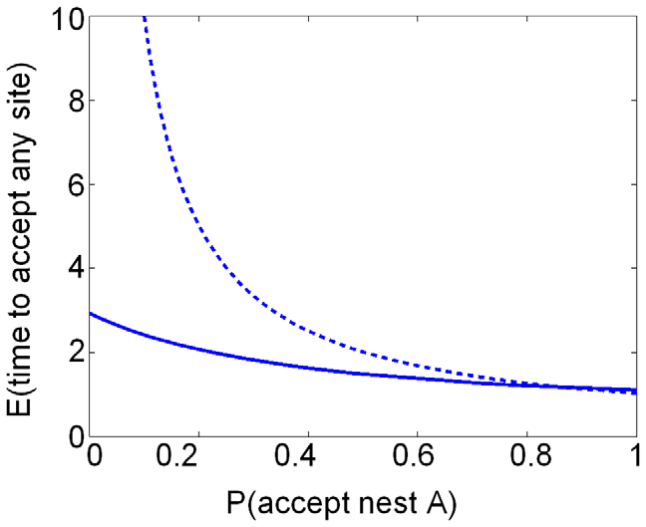
Analytical results of Markov Chain model. Expected time for an ant to accept any site across varying qualities of site A, calculated from equation 2. Dashed line: only site A present. Solid line: two nests present; site B fixed at P(accept nest B) = 0.88 (estimated from data, see Text S1, Fig. S1). For two nests present, either site is equally likely to be discovered first. Nest rediscovery probability *r* = 0.7 (estimated from data, see Text S1, Table S1). The reduced effect of site A's quality on expected decision time is robust to variations in site B's quality, except where this becomes low (see Fig. S1).

To examine analytically whether recruitment latency varies greatly with nest quality when there are two equidistant potential nest sites available, or when there is only one nest site available, we can make use of equation 2. We consider the two site case first; to do this we shall hold one of the nest site qualities constant while varying the other nest site quality, and examine the effect on the expected time to reach commitment to either site. Figure 6 illustrates how expected decision time varies for the one and two site cases, for a given revisiting value of *r* (0.7, estimated from empirical data). We vary the quality of the poor site (A) while keeping the quality of the good site (B) constant, holding *g* constant while varying *p* in the interval [0, 1] (see eq. 2). For a moderate quality of the constant nest (P(accept B) = *g* = 0.88, estimated from empirical data) expected decision time varies little between the extremes of the other nest's quality (P(accept A) = *p* = 0 to 1), thus when two nest sites are present, strong quality dependent recruitment latency is not evident (Fig. 6, solid line). This contrasts markedly with the situation where there is only one potential nest site; this is equivalent to setting *p* = *g* in equation 2. Now a much more pronounced relationship between the quality of this site, and the expected time until commitment, is evident (Fig. 6, dashed line). These analytic results agree qualitatively with results from the Monte-Carlo simulation (Fig. 5). This is good evidence for the threshold rule, but does not in itself rule out sequential comparisons since a version of the model with sequential comparisons gives almost indistinguishable results (see Text S1, Fig. S2). However, the sequential comparison model makes greater cognitive demands on individuals, who must remember the qualities of visited sites and evaluate this in making subsequent comparisons.

### Three-New-Nests: Simulation Predictions and Empirical Data

A total of 752 ants (across 6 colonies) were individually radio-tagged and their scouting activity was monitored during emigration experiments in which each colony chose between one good and two poor nests (Fig. 1c). According to the threshold rule, ants which independently (i.e. not by following a tandem run) visit the good nest (A) and one of the poor nests (designated B) and then visited another nest before quorum was reached (at which point carrying behaviour begins), should be equally likely to return to good nest A or discover the other, equidistant, poor nest (designated C). In contrast, if ants are using best-of-n direct comparisons, ants which have visited good nest A and then nest B should be more likely to return to A than to discover C.

In the empirical data, 20 individuals out of the 752 we monitored fulfilled the necessary behavioural criteria of finding A and then B before quorum was reached. This sample size is small because this behaviour was fairly rare – as would be predicted by our model, ants finding A tended to start recruiting, rather than discover B. Using this sample size of 20 ants for 100 simulation replicates gives 95% confidence intervals of 7–12 ants returning to A; 8–13 ants discovering C. The empirical data fit well within these limits, with 11 ants returning to A and 9 discovering C. This does not differ significantly from random choice (binomial test, *P* = 0.82). This supports the threshold hypothesis and shows no evidence that ants act on information from comparison of nests to allow them to preferentially return to a better nest.

Although our data show that ants did not preferentially return to the good nest, the comparison hypothesis could still be supported if those that did return to it did so more directly because they had remembered its high quality. This is not the case: ants made 0–27 (median = 3) repeat visits to B before returning to A, compared to 0–6 (median = 0) visits to B before discovering C (Mann-Whitney test: *U* = 73, *N* = 20, *P* = 0.07). The trend is in the opposite direction to that predicted by the hypothesis that ants make a comparison and act on it to return more directly to better nests. These data are consistent with threshold-based choice.

A second test of whether individual ants are comparing nests involves decisions to recruit nest-mates by leading a tandem-run to the new nest. If ants are using a ‘best-of-n’ comparison strategy, the sampling period before recruiting should be independent of the quality of the first nest they visit, whereas if ants use an internal threshold rule, the number of nests they sample before recruiting should be strongly dependent on the quality of the first nest they visit, with ants that find the high quality nest first being likely to initiate recruitment to this nest immediately. In the empirical data, 13 individual ants initiated recruitment by leading a tandem run, of which 8 had discovered the good nest first, and 5 had discovered a poor nest first. Using these sample sizes for 100 simulation replicates predicts that ants finding the good nest first should not visit any other nest before recruiting (median total nests visited: 95%CI 1-1) whereas ants finding the poor nest first should be more likely to visit other nests before recruiting (median total nests visited 95%CI 1-2). The empirical data fit these predictions. Ants which found the good nest first visited a median of only that 1 nest before recruiting, whereas those which found the poor nest visited a median of 2 different nests before leading a tandem run (Mann-Whitney test, *U* = 36, *N* = 13, *P*<0.05). The data do not support the recruitment latency mechanism, as there are no quality-dependent differences in time from nest discovery to recruitment by tandem-running (good nest: mean ± SD = 20±19 min; poor nest: mean ± SD = 28±24 min; t-test: *t*_12_ = 0.71, *P* = 0.49).

## Discussion

Our results show that a very simple individual mechanism can produce effective collective decision-making. The predictions of our threshold decision-making model correspond well with empirical data from house-hunting ants. Our simulations of the threshold rule not only predict successfully the empirical ant behaviour newly reported here, but also can explain the empirical results in two previously published studies [20], [26] under the assumption of very large differences in nest quality. This explanation of empirical data is supported by our analytical modelling results. Our results clearly support the hypothesis that this simple parsimonious mechanism (the threshold rule) is sufficient to reproduce observed empirical patterns of collective decision-making: our results can account for both apparent direct comparison of nest quality and for apparent quality-dependent recruitment latency, which had been previously proposed as mechanisms of collective choice. Our parsimonious threshold model is sufficient to explain the data without assuming more complex behavioural mechanisms. This highlights the need for carefully designed experiments to determine whether or not individuals make comparisons, and demonstrates that group-level comparison behaviour does not necessarily imply individual-level comparison behaviour. In the case of ants, evidence of the ability of lone individuals to make direct comparisons of alternatives has only recently been published [25]; as our results show, previously published studies were not sufficient to demonstrate this. Further empirical investigation is required to establish what role, if any, direct comparison by individual ants plays in the collective decision-process.

We can subdivide comparison behaviour into two types that can aid decision-making: best-of-n comparisons and sequential comparisons. Best-of-n comparison mechanisms are characterised by a sampling period, and preferential return to the best option [35]. We conducted a multiple-nest experiment to test directly if real ants show these behaviours. Neither is observed. In contrast, the results support the threshold model: if the first nest they encountered was of a high quality, the ants started recruitment immediately without continuing to sample, and those ants which had the opportunity to compare nests did not act on this information by preferential or more direct return to better nests.

Sequential comparisons occur when each time an animal encounters a new option, it compares the new option to its memory of previously encountered options and is more likely to accept the new one if it compares favourably. This is similar to the threshold model, but thresholds are influenced by experience. In one variant of this, individuals sample several options and then accept the next option they encounter which exceeds the quality of all previous options. This strategy can be shown to provide the optimal solution to the ‘secretary problem’; a sequential search problem in which previously encountered options are subsequently unavailable [36]. We modelled a sequential comparison mechanism (see Text S1) to compare with our threshold model. The results show that this type of comparison model predicts similar recruitment latency results to our threshold model. We therefore cannot rule out this sequential comparison mechanism, but note that it is a less parsimonious solution. Our threshold model is sufficient to explain the data without invoking memory and comparison during the decision process.

Our simulated ants have no memory of previously visited nests, and yet behaviour previously described as ‘comparison’ still emerges in our simulations (Table 2). This clearly shows that empirical data of this sort cannot be used as evidence that an animal is really remembering and comparing. The decision process we propose is ‘memoryless’ in the sense that individual ants do not have to remember previously visited alternatives during their search for an acceptable nest. Our model deals only with the decision process up to the point at which an ant accepts a nest. After that point, real ants clearly do use memory of nest location in order to recruit nest-mates to that nest by tandem-running [20]. Recruitment is the most easily identifiable action indicating commitment, but ants that have accepted a nest may also act on their commitment by making repeat visits to the nest [26], [37]. By making repeat visits, ants are able to assess nest area more accurately [37] and would also contribute to the accumulation of a quorum at their chosen nest, causing the switch to rapid nest-mate carrying behaviour [18]. Memory is therefore an important part of the implementation of the decision, but ants following the threshold rule do not need to invest in remembering the qualities or locations of multiple nests during the decision-making process.

The second previously-proposed mechanism, quality dependent recruitment latencies, is an attractive idea – it does not rely on comparisons, and self-organised decisions can emerge because ants finding a poor nest delay before recruiting, effectively giving others that have found a good nest a head-start in the recruitment process. The initial advantage is amplified by recruitment, and this can be shown to be sufficient to account for collective choice [24]. In both the threshold rule and the recruitment latency hypothesis, a scout makes a probabilistic, quality-dependent decision about whether to recruit each time she visits a nest. The difference between these mechanisms come from the assumptions made about what the scout does after leaving the nest, and how this results in a colony decision-mechanism. In our threshold rule, the ant either commits or continues searching. In the recruitment latency hypothesis, ants which do not immediately start recruiting may subsequently recruit, with a quality-dependent latency. In the original formulation of this idea [20] it is mentioned that ants might discover other nests during this latent period, but it is not suggested that the latency is actually a *result* of the search for other nests. Searching for alternatives has a more prominent role in later versions of the recruitment latency model [24] making it more similar to our threshold model, however, the idea of simple quality-dependent recruitment latencies functioning as a choice mechanism still appears often in discussions of collective decision-making [11], [38], [39]. There are two empirical problems with such a mechanism. Firstly, colonies are able to choose a good nest, even when it is so much further away than a poor nest that travel time should cancel out differences in recruitment latency [27]. Secondly, all empirical evidence for quality-dependent recruitment latencies comes from studies offering a colony just one new nest [20], [22], [24], [28]. When recruitment latencies are measured in multiple nest experiments, no quality-dependent recruitment latencies differences are observed [this study and 26]. We used modelling to investigate these empirical results. Our simulation and analytical results reproduce quality-dependent recruitment latency in a single-nest experiment, but show that the presence of an extra nest disrupts the quality-dependence of the latency to commitment and recruitment (Figs. 5–6). When a single poor nest is offered, acceptance times in the model will be geometrically distributed, with few ants accepting the site early due to rare assessment errors or low individual-thresholds, and the remainder continuing to re-assess the same site until they eventually decide it is good enough. When a second higher quality site is introduced, the same small proportion of ants will accept the poor site early due to low-threshold or assessment error, but the remainder will now tend to discover the alternative, superior site, and recruit to this quickly on average because of its increased quality. The same pattern would be observed for a threshold-rule with direct comparison (see Text S1), and also for a quality-dependent recruitment latency-rule, if it is assumed that ants search for alternatives during the latency period and have a high probability of finding other sites [20]. However, these alternative models are less parsimonious, requiring ants to remember the qualities and/or locations of visited sites and evaluate this in making subsequent comparisons or use the information to decide when and where to start recruiting. These results also clearly demonstrate that a phenomenon observed when only one option is available, cannot be assumed to function in the same way when choosing between multiple options (see also [7]).

Another well-studied example of collective decision-making is nest-site choice by swarming honeybees (*Apis mellifera*). Like ant colonies, bee swarms are able to choose between different nest sites, and individual bees do not need to have visited multiple sites for the swarm to be able to choose between them [40]. However, the way the scouts act on this information differs between the two groups. The ants seem to use a binary decision process to either accept that nest or continue searching. Ants accepting a nest recruit others, causing a positive feedback process, but ant scouts do not modulate their recruitment according to nest quality, e.g. by recruiting at a faster rate or for longer to a higher quality nest [20], [26]. In contrast, bees use a graded process, whereby scouts initially discovering a new nest-site almost always recruit [41], but the duration and rate of the recruiting waggle-dances are dependent on nest quality [42]. This means that more recruits are brought to better nest-sites, leading to a positive feedback process usually resulting in a unanimous choice of the better site [42]. One possible reason for this difference in strategy could be differences in nest availability: if suitable honeybee nest sites are relatively rare, then it might be better for scouts to avoid ever rejecting a potential nest outright.

Collective decisions are also made in other contexts, such as choice of foraging site. Here again, comparisons of sites by individuals seems to play little role. Foraging honeybees do not compare food sources [43], nor do they compare the waggle-dances of recruiters at the hive [44]. Rather, multiple sites ‘compete’ for foragers, with bees foraging more persistently at high quality sites and recruiting to them more energetically [43], [45]. Foragers can also directly inhibit recruitment if they perceive danger at a particular food source by the use of ‘stop-signals’ directed at bees recruiting to that source [46]. In trail-laying ants and bees, pheromone trails to better foraging sites are more strongly reinforced and this again leads to a collective choice without any necessity for individuals to visit multiple sites [47], [48]. Just as for nest-site choice, foraging recruitment can either be graded (laying more pheromone to a better food source, e.g. [47], [49]) or a binary decision (all-or-nothing recruitment, e.g. [50], [51]).

Direct comparison of alternatives is seen in a range of animals and contexts, including foraging in scrub jays and humans [8], [52], mate choice in bower-birds and dance-flies [53], [54] and shell assessment by hermit crabs [55]. A best-of-n comparison strategy would maximise decision accuracy [2], so why do neither individual bees nor ants appear to use this strategy when foraging or choosing a new home? One possibility is that multiple comparisons are too cognitively complex for ant and bee brains. However, small insects including bees are competent at related cognitive processes, including contextual learning, discrimination between stimuli and associative recall [56]. Male dance-flies compare the size of potential mates before choosing [54], and there is evidence to suggest that honeybee workers are capable of comparative evaluation of flowers [57] and that lone *T. rugatulus* ant workers are capable of comparing the attributes of nest-sites which are very close together [25]. There may, therefore, be reasons other than cognitive constraints for why relying on individual best-of-n comparisons to inform collective choices does not appear to have evolved as a decision mechanism in bees or ants. One possible reason is that during their evolutionary history the ants have typically been faced with decisions about whether to accept a single option at a time, rather than simultaneously having information about multiple options, and this has shaped the evolution of their decision process [7]. Another possible reason is the potential speed advantage of avoiding a sampling period. Sampling periods can be costly [2], [7] and pose the mathematically complex problem of deciding how long to sample for before starting the decision process [58]. Avoiding best-of-n comparisons allows a potentially vulnerable colony to select the first nest encountered, if it is good enough. Perhaps even more important is distributing the decision-making process, relying less on information-processing by a single individual; rather allowing the decision to emerge from a collective process to which even partly-informed individuals can contribute. In this colony-level cognition, the relevant information about the environment is represented within the individuals in a colony, their actions and interactions. At the collective level this generates new information: which nest the colony should choose [59]. In both bees and ants, the information held by individuals is integrated into a colony choice by means of a quorum threshold, which triggers rapid implementation of the decision [18], [60]. Although the colony will usually choose the site which reaches quorum first, this does not mean that the colony as a whole must ‘satisfice’, accepting the first nest surpassing some minimum standard. Using quality-dependent initiation (in *Temnothorax* ants) or modulation (in honeybees) of the recruitment process leading to the attainment of a quorum, the colony in effect carries out a concurrent but indirect collective comparison process, which may implement optimal decision-making through the actions of partly-informed individuals [61]. This ensures that the nest to reach a quorum first is also likely to be the best option, even when there is an array of possible alternatives [16], [42]. Thus without the individuals using a ‘best-of-n’ comparison strategy, the colony is able to solve a ‘best-of-n’ challenge [16], [62].

A further possible advantage of avoiding individual direct comparisons is that it would remove the risk of ‘irrational’ decision behaviour at the individual level [26], associated with comparative, relative evaluation of options such as best-of-n, or sequential comparisons. Individuals following our proposed threshold rule make an absolute evaluation of quality, and this evaluation of one option is not influenced by the other alternatives available, in contrast to the context-dependent decision-making used by many other animals including humans [8], [63], [64]. Although at the collective level, a comparison between options is effectively being made, a non-comparative individual-level mechanism for this decision could protect the colony from irrationality in decision-making. For example, emigrating *T. albipennis* colonies follow rational transitivity patterns in decision making [16] and in the related species *T. curvispinosus* and *T. rugatulus*, colonies seem to be immune to irrational distractor effects from irrelevant alternatives [25], [65]. Although work on *T. rugatulus* suggests that this collective immunity can emerge even if individuals are susceptible to distractor effects provided their individual contribution to the collective decision is small [25], our work suggests this collective rationality can emerge without depending on low numbers of ants visiting both nests – if individual ants follow the threshold rule, then the colony can escape these irrational comparison behaviours.

Using an absolute evaluation method does have a potential drawback, if the acceptance threshold is not appropriate to the environment. When used in individual decision making (e.g. during mate choice in cockroaches, [9]) the success of this sort of sequential search strategy will be highly sensitive to the threshold used. For example, if all available options are poor quality, an animal may reject them all, and end up worse off than if it had accepted a poor option. In contrast, when this strategy is used for group decision making, variation in acceptance thresholds across the group provides protection against this sensitivity. If all options are poor quality, some low-threshold individuals will still accept one, allowing the group to make a choice. Similarly, intra-colony variation in task response thresholds promotes effective division of labour in social insect colonies, with more individuals engaging in a particular task when the stimulus for that task increases [66]–[68]. Our model assumes that individual thresholds are fixed over the time-scale of the emigration process, however further flexibility would be available if acceptance thresholds were updated based on experience over the course of an individual's life-time [69], [70]. Flexible task thresholds promote flexible division of labour in social insect colonies [71]–[73]. How much acceptance threshold variation actually occurs within a colony, and whether ant acceptance thresholds are fixed throughout life or changed by experience, are issues remaining to be explored.

In summary, our simple individual-threshold model is sufficient to explain empirical decision-making data, without assuming more complex behavioural mechanisms. Since the scientific method gives preference to the more parsimonious model, this result shows that care is needed in inferring individual behaviour from group behaviour. We propose that, in general, individual decision-makers can use this simple threshold rule to provide their group with an elegant solution to the problem of making a collective choice. This strategy allows the group to make a rational, well-informed and rapid decision.

## Supporting Information

Figure S1 **‘Expected decision time in a two-nest scenario.** Expected time for an ant to accept any site across varying qualities (probabilities of acceptance) of sites A and B, calculated from equation 2.
(TIF)

Figure S2 **‘Expected decision time in one-nest scenario.** Expected time for an ant to accept any site, using the parameters of Figure 6, for the no-comparison threshold-rule of the main text (red), and for the direct-comparison threshold-rule described above (green).
(TIF)

Text S1 **This supplementary model information covers details of model parameterisation for the analytical and simulation modelling.** It also describes an alternative analytic model of thresholds with sequential-comparison. It provides the basic model code of the Monte Carlo model.
(DOC)

Table S1 **Further details of model parameterization.**
(PDF)
