# Supplementary figures and images for: A Simple Threshold Rule Is Sufficient to Explain Sophisticated Collective Decision-Making
Source: PLoS One. 2011 May 24;6(5):e19981. doi: 10.1371/journal.pone.0019981 (PMC3101226; doi:10.1371/journal.pone.0019981)

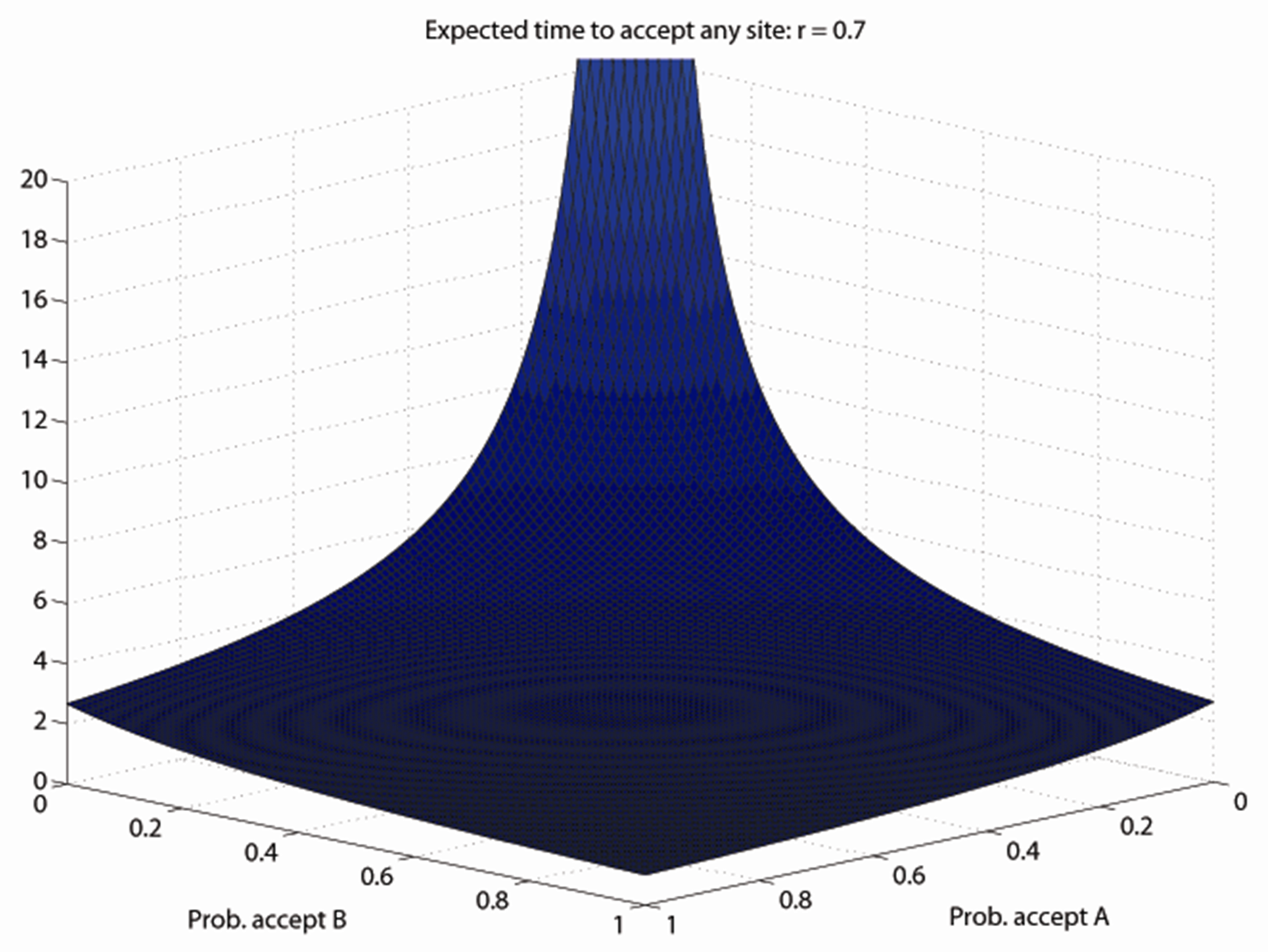

Supplement: Figure S1 — ‘Expected decision time in a two-nest scenario. Expected time for an ant to accept any site across varying qualities (probabilities of acceptance) of sites A and B, calculated from equation 2. (TIF) [file pone.0019981.s001.tif]

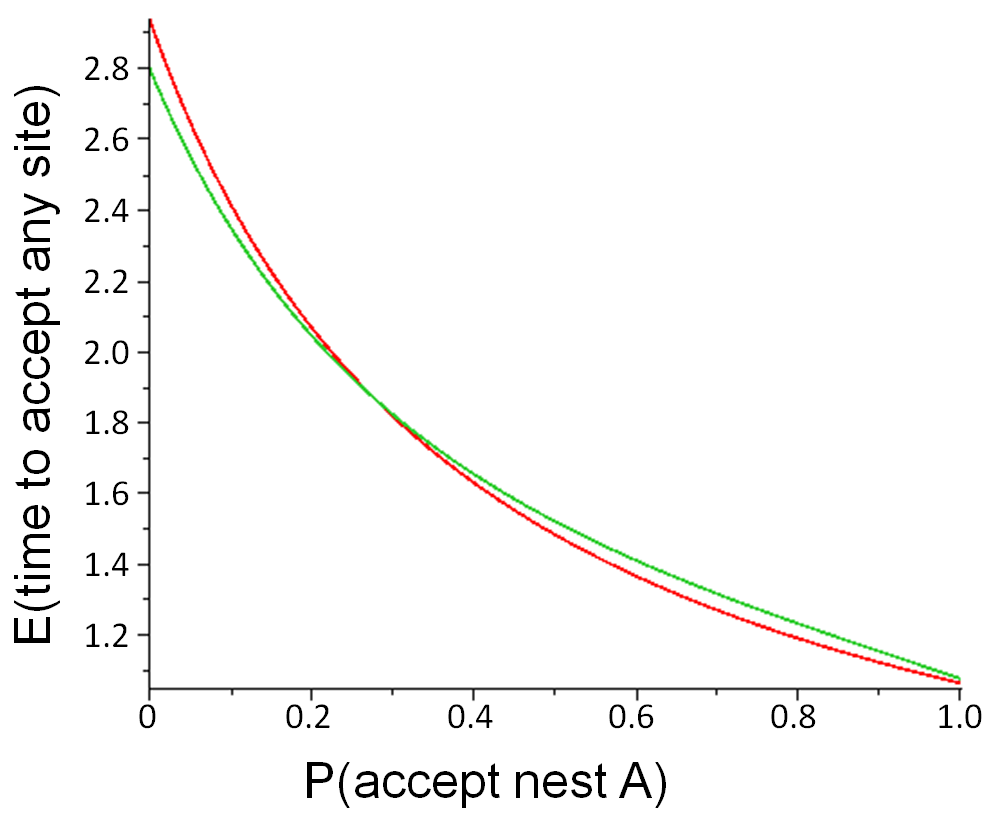

Supplement: Figure S2 — ‘Expected decision time in one-nest scenario. Expected time for an ant to accept any site, using the parameters of Figure 6, for the no-comparison threshold-rule of the main text (red), and for the direct-comparison threshold-rule described above (green). (TIF) [file pone.0019981.s002.tif]
