# Supplementary material for: A Simple Threshold Rule Is Sufficient to Explain Sophisticated Collective Decision-Making
Source: PLoS One. 2011 May 24;6(5):e19981. doi: 10.1371/journal.pone.0019981 (PMC3101226; doi:10.1371/journal.pone.0019981)
Supplement: Text S1 — This supplementary model information covers details of model parameterisation for the analytical and simulation modelling. It also describes an alternative analytic model of thresholds with sequential-comparison. It provides the basic model code of the Monte Carlo model. (DOC) [file pone.0019981.s003.doc]

**TEXT S1: SUPPLEMENTARY MODEL INFORMATION**

**Parameterisation of discovery probabilities**

The probability that a searching ant finds a particular nest depends not only on the relative positions of those nests, but also on the size and shape of the whole arena in which the ant is searching.

We used a NetLogo [1] simulation to generate discovery probabilities. We created environments with the shape and dimensions used in the experiments (Table S1), The search patterns of ants were described using two distributions (step lengths and turn angles) which were generated from video data of follower *T. albipennis* ants searching for a removed leader during tandem running [2]. Each simulated ant left the old nest in the direction the entrance was pointing (Table S1) and continued for the length of a step chosen at random from the step-length distribution. It then changed angle of heading by an amount chosen randomly from the turn-angle distribution and made a new step. If an ant encountered a wall part way through a step, its route for the rest of that step was deflected to follow the wall in the direction which required least change of angle. This is consistent with ant wall-following behaviour. When the ant came within 4 mm of a nest entrance, it was considered to have discovered the nest, and its turn finished. This was repeated to determine the proportion of ants which discovered each nest. Sufficient replicates were used to generate discovery probabilities repeatable to 2 decimal places (16000 replicates). The process was then repeated with the ants starting from nest A, and so on.

Note that to allow for the possibility that search patterns of lost tandem-run followers are not a good model for house-hunting scouts, we also re-ran the NetLogo simulations using a pattern of Brownian motion of the ants. Here ants which encountered walls were deflected with equal-angle. Re-running the Monte-Carlo simulations using these re-discovery probabilities does not qualitatively change the results, showing that our results are not reliant on the specific search pattern used.

**The analytical model approximates recruitment latency**

Recruitment latency in the experiments and in the simulations is defined as time between first visit to a site, and recruitment to that site. In analysing the Markov-Chain model (eq. 1) we would ideally prefer to calculate the expected value of precisely the same quantity for each of the nest sites (e.g. Fig. 5). However, this is in general a very difficult problem, so instead we use standard techniques to calculate the expected time to reach commitment to *any* site, from each of the initial starting states (assessing site A or assessing site B). Since we assume each site is equally likely to be discovered first, we therefore take the unweighted mean of these two expectations to derive the overall expected recruitment latency (eq. 2). This is an acceptable simplification; when both sites are of comparable quality, the recruitment latency (as defined above) will be roughly equal for both sites, so is well approximated by the overall expected recruitment latency (eq. 2). When one site is substantially better than the other (e.g. Fig. 6), the recruitment latency to the superior site dominates in the overall expected recruitment latency, since most ants end up recruiting to that site, and since those ants recruit quicker on average because the site is superior. In contrast, those ants that do recruit to the poorer site will be comparatively rare, and will have shorter recruitment latencies on average since those that do not accept early will tend to become committed to the superior site instead. Thus the pattern of recruitment latency dependence on site quality for the two different sites should be broadly comparable to each other and to the overall expected recruitment latency calculated in equation 2. This agrees with the simulation and empirical results presented in Figure 5.

**Parameterisation of the analytical modelling**

The analytical modelling presented in figure 6 required values for the probability an ant rediscovers the same nest (*r*), and for the probability of accepting a good nest when it is encountered (*g*).

The analytical model corresponds to case 2 above: two new nests, each equally likely to be discovered first. The probability of ants in nest A or B re-discovering the same nest is 0.70 (Table S1), so in the analytical modelling *r* = 0.7.

To calculate the probability of an ant accepting a good nest, we returned to the original case 2 data [3], see Table S1. All ants independently entering the good nest were considered. If their next act was to recruit to the good nest by carrying or tandem running, they are considered to have accepted the good nest. If their next act was to enter the poor nest they are considered not to have accepted the good nest. Other behaviours (following tandem runs and being carried) are ignored, as these do not indicate level of commitment to encountered nests. Over the three colonies for which this data is reported, a mean of 0.88 ants encountering the good nest accept it, so in the analytical modelling P(accept B) = *g* = 0.88. The reduced effect of site A's quality on expected decision time is robust to variations in *g*, except where this becomes small (Figure S1).

**Equivalent analytic model parameters for Monte Carlo model**

As discussed in the main text, parameters for the analytic model that are equivalent to those used in the Monte Carlo model can easily be calculated. The calculation proceeds on the basis of a normal distribution of acceptance thresholds with mean *μ_accept_* and variance *σ^2^_accept_*, and assessment distributions for poor and good nests with means *μ_poor_* (respectively *μ_good_*) and variances *σ^2^_poor_* (respectively *σ^2^_good_*). For the example of an ant evaluating a good nest, the probability of that ant accepting that site is given by

$g=\text{P}(\text{accept good site)=P}\left(s\sim N\text{(}\mu_{good},\sigma^{2}_{good})>t\sim N\text{(}\mu_{accept},\sigma^{2}_{accept})\right)$,

which is equivalent to

$g\text{=P}\left(u\sim N\text{(}\mu_{good}-\mu_{accept},\sigma^{2}_{good}+\sigma^{2}_{accept})>0\right)=\frac{1}{2}\left(1-\text{erf}\left(\frac{0-\mu_{good}+\mu_{accept}}{\sqrt{2(\sigma^{2}_{good}+\sigma^{2}_{accept})}}\right)\right)$,

with an equivalent equation for *p* = P(accept poor site). For Monte Carlo simulation parameters *μ_accept_* = 5, *μ_poor_* = 4, *μ_good_* = 6 and *σ^2^_accept_* = *σ^2^_poor_* = *σ^2^_good_* = 1, the above calculation gives P(accept good site) = 0.76025, and P(accept poor)=0.23975. That is, in the Monte Carlo simulations, the good site is approximately 3 times more likely to be accepted by an assessing ant than is the poor site.

**An analytic model of thresholds with sequential-comparison**

For comparison with the threshold-switching model without direct comparisons (eq. 1) we also analysed a threshold-switching model where individuals with experience of both nest sites integrate this evidence into their acceptance threshold when accepting a particular nest site. If such an informed scout does not accept the site it is currently assessing, it searches for a new site using a random walk, as in the no-comparison version of the model. Thus for an uninformed individual (i.e. without experience of any alternatives) assessing site X, the probability of acceptance is a function only of X’s quality, *f*(*x*). For an informed individual assessing current site X, but also informed of alternative site Y, the probability of accepting X is a function of X and Y’s quality, *f*(*x,y*). Since the probability of accepting site X over site Y must be the complement of the probability of accepting site Y over site X, we must have that *f*(*x,y*) = 1 – *f*(*y,x*). Then the Markov Chain model is

| $\left(\begin{matrix} (1-f(a))r & 0 & 0 & 0 & 0 & 0 \\ 0 & (1-f(b))r & 0 & 0 & 0 & 0 \\ 0 & (1-f(b))(1-r) & (1-f(a,b))r & f(a,b)(1-r) & 0 & 0 \\ (1-f(a))(1-r) & 0 & (1-f(a,b))(1-r) & f(a,b)r & 0 & 0 \\ f(a) & 0 & f(a,b) & 0 & 1 & 0 \\ 0 & f(b) & 0 & 1-f(a,b) & 0 & 1 \end{matrix}\right)$, | (S1) |
| --- | --- |

where states are respectively (from left to right, and top to bottom): Informed of A and assessing A, informed of B and assessing B, informed of A and B but assessing A, informed of A and B but assessing B, committed to A, and committed to B.

To evaluate the dependence of latency on nest qualities in the two-nest situation, as done for Figure 6, we must choose a comparison function *f*(*x,y*). The sigmoid function

| $\frac{1}{1+e^{6(y-x)}}$, | (S2) |
| --- | --- |

has the desirable property that probability of acceptance = 0.5 when both sites are of equal quality (*x*=*y*), and also the required property (above) that *f*(*x,y*) = 1 – *f*(*y,x*). The 6 in the denominator eq. S2 is a constant scaling factor ensuring that when the nests are of maximally different quality *x*=0 and *y­*=1 (or vice versa) the probability of accepting the current site is sufficiently close to 0 (or 1).

Analysing for the expected overall recruitment latency using the same parameters as in Figure 6 yields a much more complicated expression than eq. 2 (not shown), but plotting the two against each other reveals quantitatively very similar behaviour (Figure S2). Hence a direct-comparison threshold-based model reproduces the same latency patterns as the non-comparison threshold-based model, and as seen in the empirical data (Figs 5 & 6), so these patterns do not rule out a sequential comparison mechanism.

**Supplementary Information: Model code**

The simulation model was implemented in MatLab 7.4.0. The code for the basic model is reproduced below.

function [times, preqtimes, discovers, preqdiscovers, visits, preqvisits, accepts, preqaccepts] = assessEJHR5(n, quals, probs, threshold_mean, threshold_stddev, quality_stddev, time_means, time_stddevs, quora)

% n = number of replicates (>=1)

% qualities = row vector of m site qualities

% (qualities(1) = home site

% quality: -Inf for no effect of home site quality on searching)

% discovery_probabilities = m * m matrix of discovery probabilities from

% column site to row site (N.B. columns should sum to 1)

% threshold_mean: mean population threshold for site acceptability

% threshold_stddev: standard deviation in population thresholds

% quality_stddev: standard deviation in quality assessments (eta)

% time_means: m * m matrix of mean travel times from column site to row

% site (N.B. should probably be symmetric)

% time_stddevs: m * m matrix of travel time standard deviations, from

% column site to row site (N.B. should probably be symmetric)

% quora: 1 * m matrix of quorum times for each nest site

%

% times = row vector of times to first recruitment (i.e. nest acceptance)

% discovers = matrix (m x i) of times of first visit to each site

% visits = matrix (m x i) of numbers of visits to each site

% accepts = row vector of ids of accepted sites (indexed from 1 (for home nest) to m)

% the equivalents prefixed 'preq' are the pre-quorum equivalents of these

for i=1:n

% Monte Carlo simulation of one ant

times(i) = 0;

preqtimes(i) = 0;

accepts(i) = 1; % ant is in home site

preqaccepts(i) = 1;

discovers(1,i) = -1; % ant is already in home site

preqdiscovers(1,i) = -1;

visits(1,i)=1; % ant is already in home site

preqvisits(1,i)=1;

for j=2:(size(quals,2))

discovers(j,i) = 0; % ant has not discovered or visited other sites

visits(j,i) = 0;

preqdiscovers(j,i) = 0;

preqvisits(j,i) = 0;

end

thresh = normrnd(threshold_mean, threshold_stddev); % sample ant's acceptance threshold

while thresh > normrnd(quals(accepts(i)), qual_stddev)

% search

ran = unifrnd(0,1);

newsite = 1;

while ran > probs(newsite, accepts(i)) %finds one of the available sites

ran = ran - probs(newsite, accepts(i));

newsite = newsite + 1;

end

delta = max(1, normrnd(time_means(newsite, accepts(i)), time_stddevs(newsite, accepts(i)))); % normally-distributed time-step size (>=0)

times(i) = times(i) + delta;

accepts(i) = newsite;

if discovers(newsite,i)== 0

discovers(newsite,i)= times(i);

end

visits(newsite,i)=visits(newsite,i)+1;

if times(i) > quora (1, newsite) %if past pre-quorum period, then no new nests can be added

preqtimes(i) = NaN;

preqaccepts(i) = preqaccepts(i);

if preqdiscovers(newsite,i)== 0

preqdiscovers(newsite,i)= NaN;

end

preqvisits(newsite,i)=preqvisits(newsite,i);

else

preqtimes(i)=times(i);

preqaccepts(i) = newsite;

if preqdiscovers(newsite,i)== 0

preqdiscovers(newsite,i)= times(i);

end

preqvisits(newsite,i)=preqvisits(newsite,i)+1;

end

end

% recruit / accept nest

end

end

**Supplementary References**

1. Franks NR, Richardson TO, Keir S, Inge SJ, Bartumeus F, et al. (2010) Ant search strategies after interrupted tandem runs. J. Exp. Biol. 213: 1697-1708.

2. Mallon EB, Pratt SC, Franks NR (2001) Individual and collective decision-making during nest site selection by the ant *Leptothorax albipennis*. Behav Ecol Sociobiol 50: 352-359

3. Wilensky, U. 1999. NetLogo. <http://ccl.northwestern.edu/netlogo/>. Center for Connected Learning and Computer-Based Modeling, Northwestern University. Evanston, IL.

**Figure legends for Supplementary Figures**

**Figure S1. 'Expected decision time in a two-nest scenario.** Expected time for an ant to accept any site across varying qualities (probabilities of acceptance) of sites A and B, calculated from equation 2.

**Figure S2. 'Expected decision time in one-nest scenario.** Expected time for an ant to accept any site, using the parameters of Figure 6, for the no-comparison threshold-rule of the main text (red), and for the direct-comparison threshold-rule described above (green).
