# Supplementary material for: A Simple Threshold Rule Is Sufficient to Explain Sophisticated Collective Decision-Making
Source: PLoS One. 2011 May 24;6(5):e19981. doi: 10.1371/journal.pone.0019981 (PMC3101226; doi:10.1371/journal.pone.0019981)
Supplement: Table S1 — Further details of model parameterization. (PDF) [file pone.0019981.s004.pdf]

Table S1. The parameterisations of nest re-discovery probabilities, using empirically-derived ant search patterns in Net Logo simulations.

	**Case 1: Good nest far, poor nest near [1]**	**Case 2: Two equidistant nests [2]**	**Case 3: Three equidistant nests (new experiment)**
Arena shape and dimensions	Rectangle:<br>180 x 1800 mm	Rectangle:<br>750 x 430 mm	Circle:<br>radius 500 mm
Positions of entrance to each nest (as mm grid coordinates with old nest at origin	Old = (0,0)<br>A = (0, 300)<br>B = (0, 1200)	Old: (0,0)<br>A: (340, 300)<br>B: (-340, 300)	Old= (0,0)<br>A= (0, 386)<br>B= (334, -193)<br>C= (-334, -193)
Nest entrances orientation	Old: 0º<br>A: 180º<br>B: 180º	Old: 0º<br>A: 180º<br>B: 180º	Old: Random[1]<br>A: 180º<br>B: 300º<br>C: 60º
Probabilities of finding nests (from column to row)	Old A B<br>Old {0.91 0.15 0.03}<br>A {0.06 0.80 0.06}<br>B {0.03 0.05 0.91}	Old A B<br>Old {0.70 0.15 0.15}<br>A {0.15 0.70 0.15}<br>B {0.15 0.15 0.70}	Old A B C<br>Old{0.76 0.08 0.08 0.08}<br>A {0.08 0.82 0.05 0.05<br>B {0.08 0.05 0.82 0.05}<br>C {0.08 0.05 0.05 0.82}

1.Robinson, E. J. H., Smith, F. D., Sullivan, K. M. E. & Franks, N. R. 2009 "Do ants make direct comparisons?" Proc. R. Soc. Lond. B. 276, 2635-2641. (doi:10.1098/rspb.2009.0350)
2. Mallon, E. B., Pratt, S. C. & Franks, N. R. 2001 "Individual and collective decision-making during nest site selection by the ant *Leptothorax albipennis*." Behav. Ecol. Sociobiol. 50, 352-359. (doi:10.1007/s002650100377)

[1] This nest had a central exit in the roof, so no bias in direction of leaving would be introduced
